# Gd^3+^ engineered Co–Mn–Mg spinel nanoferrites for multifunctional theranostics: magnetic hyperthermia, antioxidant hepatoprotection, and antibacterial activity

**DOI:** 10.1039/d5ra08039h

**Published:** 2025-12-08

**Authors:** M. Elansary, N. Bentarhlia, O. Oulhakem, Y. Mouhib, B. Salameh, A. M. Alsmadi, B. Kartah, H. Monfalouti, M. Belaiche, O. M. Lemine

**Affiliations:** a Nanoscience and Nanotechnology Unit, E.N.S Rabat, Energy Research Centre, Mohammed V University B.P. 5118 Takaddoum Rabat Morocco moustapha_bouzegou@um5.ac.ma; b Center for Graphene Research & Innovation, University of Mississippi MS 38677-1848 USA; c College of Sciences, Department of Physics, Imam Mohammad Ibn Saud Islamic University (IMSIU) Riyadh 11623 Saudi Arabia; d Laboratory of Plant Chemistry, Organic and Bioorganic Synthesis, Faculty of Sciences, Mohammed V University in Rabat Morocco; e Department of Physics, Kuwait University Safat 13060 Kuwait; f Laboratory of Molecular Chemistry, Materials and Catalysis (LC2MC), Faculty of Science and Technology of Béni-Mellal, University Sultan Moulay Slimane B.P. 523 Béni-Mellal 23000 Morocco

## Abstract

We synthesized Co_0.5_Mn_0.25_Mg_0.25_Fe_2−*x*_Gd_*x*_O_4_ (*x* = 0.00; 0.04; 0.06) nanoferrites using a sol–gel auto-combustion method and studied their structural, magnetic, and biological properties. XRD with Rietveld refinement confirmed the formation of a pure spinel structure with nanosized crystallites. FTIR and XPS analyses proved the presence of metal–oxygen bonds, mixed oxidation states of Fe and Co, and the successful incorporation of Gd^3+^. TEM images revealed nanometric particles with homogeneous elemental distribution. Magnetic measurements showed that Gd^3+^ doping modifies the saturation magnetization (*M*_s_) and coercivity (*H*_c_), with the best performance at *x* = 0.04 (*M*_s_ = 45.7 emu g^−1^, *H*_c_ = 427 Oe). Under an alternating magnetic field, the samples efficiently produced heat in the hyperthermia range, with a specific absorption rate (SAR) of about 34 W g^−1^ for *x* = 0.04. *In vivo* experiments in ethanol-induced liver injury models demonstrated that the *x* = 0.04 sample improved antioxidant activity (increased SOD and CAT levels) and restored important serum biochemical markers such as albumin, total protein, creatinine, urea, uric acid, and electrolytes. This indicates strong hepatoprotective and nephroprotective effects. Antibacterial studies further showed that the nanoferrites were more effective against Gram-positive bacteria (*S. aureus*, *B. subtilis*, *B. licheniformis*) than Gram-negative ones (*E. coli*, *P. aeruginosa*). Overall, our results show that Gd^3+^ substitution enhances both magnetic and biological properties. The *x* = 0.04 composition provides the best compromise between magnetic heating efficiency, antioxidant protection, and antibacterial activity, making these nanoferrites promising candidates for biomedical applications such as cancer hyperthermia therapy, antioxidant defense, and infection control.

## Abstract 2

We synthesized Co_0.5_Mn_0.25_Mg_0.25_Fe_2−*x*_Gd_*x*_O_4_ (*x* = 0.00; 0.04; 0.06) nanoferrites using a sol–gel auto-combustion method and studied their structural, magnetic, and biological properties.

## Graphical abstract

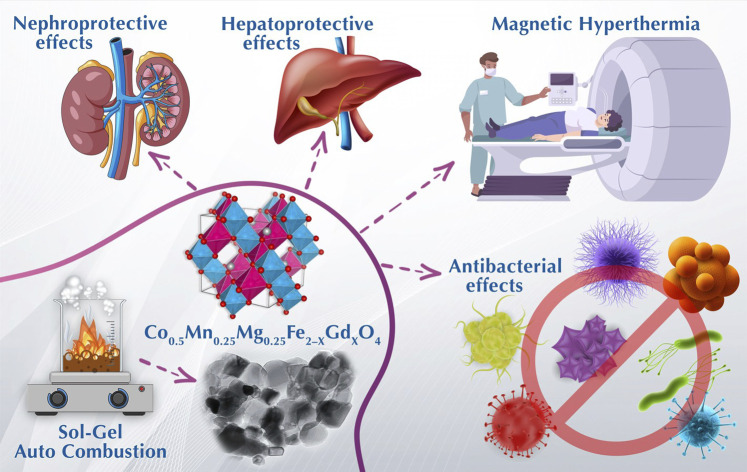

## 1 Introduction

Magnetic nanoparticles based on spinel ferrites remain at the forefront of interdisciplinary research due to their tunable physicochemical properties and their applications in electronics, catalysis, and biomedicine.^1,2^ Ferrites with the general formula MFe_2_O_4_ (M = Co, Mn, Ni, Zn, …) offer a wide range of magnetic behaviors that can be controlled by composition, particle size, and cation distribution between the tetrahedral (A) and octahedral (B) sites of the spinel lattice.^3^ These structural aspects directly govern the saturation magnetization (*M*_s_), coercivity (*H*_c_), anisotropy, and magnetic losses critical properties for applications ranging from MRI to magnetic hyperthermia.^4,5^

Among ferrites, cobalt ferrite (CoFe_2_O_4_) is widely studied due to its strong magnetocrystalline anisotropy and thermal and chemical stability.^6–8^ However, its intrinsic magnetic parameters (high *H*_c_, variable magnetization) may be unsuitable for certain biomedical applications that require a compromise between magnetic performance and biocompatibility (*e.g.*, hyperthermia); hence the interest in cation doping to finely tune these properties.^9,10^

Doping with transition ions (Mn^2+^, Mg^2+^, *etc.*) or rare earth ions (Gd^3+^, Ce^3+^, …) is a well-established strategy to modify the cation distribution, crystallite size, and local anisotropy.^11^ The introduction of Mn^2+^ into CoFe_2_O_4_ often tends to increase or restructure the effective magnetic moment and to modify the coercivity through A/B site redistribution, while dopants such as Gd^3+^ (4f ions) can strongly influence local anisotropy, exchange interactions, and relaxation dynamics parameters that are crucial for hyperthermia performance and for surface properties involved in biological applications.^12–15^

Magnetic hyperthermia (magnetic fluid hyperthermia, MFH) utilizes the energy dissipation (specific absorption rate, SAR) of nanoparticles subjected to an alternating magnetic field to locally raise the tumor temperature (42–46 °C).^16–18^ The SAR efficiency strongly depends on size, shape (spherical *vs.* cubic/nanocubes), colloidal dispersion, *M*_s_, *H*_c_, and the frequency/amplitude of the field. Recent publications have demonstrated significant SAR gains by optimizing morphology (nanocubes) and composition (tailored doping), making some formulations close to potential clinical translation if safety and biodistribution are satisfactory.^19–22^

From a synthesis perspective, the sol–gel auto-combustion method offers a versatile and efficient route for producing nanometric ferrites with precise stoichiometric control and homogenous cation distribution. This technique enables fine tuning of particle size, morphology, and crystallinity by adjusting parameters such as the fuel-to-oxidizer ratio, pH, and calcination temperature. The inherent combustion process leads to rapid formation of porous, loosely agglomerated powders with high surface area, beneficial for subsequent functionalization. Recent advancements in sol–gel protocols focus on improving particle stability and surface modification to enhance biocompatibility and optimize *in vivo* behavior, which are critical factors for biomedical applications including hyperthermia and targeted drug delivery.

Beyond applications in hyperthermia and MRI, doped ferrites also show promising uses in catalysis (notably for pollutant degradation),^23^ nanomedicine (as antioxidants and potential hepatoprotective agents *via* modulation of redox and inflammatory pathways), as well as in energy devices.^24,25^ Dopants such as Gd^3+^ and other rare earth elements can also impart biological and imaging properties (MRI contrast agents), while raising significant concerns related to toxicity and long-term elimination. This highlights the importance of comprehensive *in vivo* evaluations, including bioaccumulation, renal function, hepatic assessment, and oxidative stress.^25,26^

The main motivation of this work is to enhance the magnetic and biological properties of spinel-type ferrites by combining multiple metallic cations. Conventional ferrites such as CoFe_2_O_4_, although stable and strongly magnetic, exhibit high coercivity that limits their use in biomedical applications. To overcome these limitations, Mn^2+^, Mg^2+^, and Gd^3+^ ions were introduced to modify the cation distribution between tetrahedral and octahedral sites, adjust the magnetization, and optimize hyperthermia performance. The incorporation of Gd^3+^, which possesses a high magnetic moment and good biocompatibility, is also intended to enhance the antioxidant and antibacterial activities of the nanoparticles. In this study, we synthesized Co_0.5_Mn_0.25_Mg_0.25_Fe_2−*x*_Gd_*x*_O_4_ nanoparticles (*x* = 0.00, 0.04, 0.06) using a sol–gel auto-combustion method. Our objective is to elucidate the correlations between structure, composition, magnetic properties, and hyperthermia performance (SAR), while simultaneously evaluating the *in vivo* hepatoprotective activity and the modulation of redox and biochemical markers. This integrated approach provides valuable insights into the therapeutic potential and biosafety profile of these multifunctional nanoferrite formulations, highlighting their promise for advanced theranostic applications.

## 2 Experimental details

### 2.1 Development of Mn–Mg–Co ferrite nanoparticles

Nanoparticles of Co_0.5_Mn_0.25_Mg_0.25_Fe_2−*x*_Gd_*x*_O_4_ (*x* = 0.00, 0.04, 0.06) were synthesized by the sol–gel auto-combustion method using metal nitrates as precursors. Analytical grade cobalt nitrate Co(NO_3_)_2_·6H_2_O, manganese nitrate Mn(NO_3_)_2_·4H_2_O, magnesium nitrate Mg(NO_3_)_2_·6H_2_O, iron nitrate Fe(NO_3_)_3_·9H_2_O, and gadolinium nitrate Gd(NO_3_)_3_·6H_2_O were used as metal sources. Stoichiometric amounts of these nitrates, corresponding to the desired compositions, were dissolved in distilled water under constant stirring to form a clear solution. Citric acid was added as a complexing agent at a molar ratio of 1.5 : 1 (citric acid to total metal ions) to chelate the metal ions and promote gel formation. The solution pH was adjusted to 7 using ammonia solution to facilitate gelation. The mixture was then heated at 80–90 °C under continuous stirring to evaporate the solvent and promote gel formation. As the solvent evaporated, the gel underwent auto-ignition combustion, producing a voluminous and loose ash-like powder. This powder was collected and subsequently calcined at 500 °C for 4 hours in air to improve crystallinity and remove organic residues, resulting in the formation of Co_0.5_Mn_0.25_Mg_0.25_Fe_2−*x*_Gd_*x*_O_4_ nanoparticles.

### 2.2 *In vivo* studies

#### 2.2.1 Materials and methods

The hepatoprotective effects of Co_0.5_Mn_0.25_Mg_0.25_Fe_2−*x*_Gd_*x*_O_4_ (*x* = 0.06) were evaluated in a rat model of liver injury induced by ethanol toxicity. Wister Rats were administered Co_0.5_Mn_0.25_Mg_0.25_Fe_2−*x*_Gd_*x*_O_4_ (*x* = 0.06) at doses of 10 mg kg^−1^ and 5 mg kg^−1^, with silymarin used as a reference standard. Hepatoprotective activity was assessed by measuring various serum biochemical markers, including liver enzymes (aspartate aminotransferase (ASAT), alkaline phosphatase (ALP)), proteins (albumin (ALB), total protein (TP)), and other indicators (total bilirubin, creatinine, urea, uric acid, triglycerides, glucose, cholesterol). Additionally, electrolyte levels (sodium, potassium, calcium, magnesium). Antioxidant enzyme levels (superoxide dismutase (SOD), catalase (CAT), and glutathione peroxidase (GPx)) were also measured. Liver histopathology was performed to evaluate liver damage.

#### 2.2.2 *In vivo* investigation of hepatoprotective effects

##### 2.2.2.1 Animals

For this study, adult Wistar albino rats (8–10 weeks old, both sexes) were sourced from the animal house of the Faculty of Sciences in Rabat. The rats, weighing between 120 and 180 ± 20 grams, were selected to ensure experimental consistency. They were housed in specially designed plastic cages under controlled conditions: temperature (25 ± 2) °C, a 12-hour light/dark cycle, and 50% relative humidity. The rats had ad libitum access to standard rat chow and water. All experimental procedures and animal handling adhered to the European Union animal care directives (EEC Council 86/609). The research protocol was meticulously reviewed and approved by the animal ethics committee of the Faculty of Sciences in Rabat, ensuring the highest standards of ethical treatment and animal welfare.

##### 2.2.2.2 Experimental design for assessing hepatoprotective effects in Adult Wistar albino rats

Adult Wistar albino rats, randomly divided into five groups (six rats per group), were subjected to ethanol-induced toxicity. After one week of adaptation to a standard diet, 16- to 17-week-old rats were assigned to the following groups: control, ethanol-exposed, positive control (receiving silymarin at 100 mg kg^−1^ body weight), and two extract treatment groups (5 mg kg^−1^ and 10 mg kg^−1^ body weight). Following the protocol described by Yi-Wei Cao *et al.* (2014) with modifications,^27^ all groups except the control were fed a liquid diet containing 11% carbohydrates, 18% protein, and 35% fat, and 36% ethanol. Silymarin and extracts were added just before mixing using a blender. This methodology was designed to assess the efficacy of silymarin and extracts in preserving liver health while adhering to stringent ethical standards for animal experimentation.

##### 2.2.2.3 Evaluation of serum biochemical markers

Blood samples were collected into tubes and centrifuged at 3000 rpm at 5 °C for 15 minutes. These samples were analyzed using an automated chemistry analyzer to measure various parameters, including Alanine aminotransferase (ALAT) (U/L), aspartate aminotransferase (ASAT) (U/L), alkaline phosphatase (ALP) (U/L), albumin (ALB) (g dL^−1^), total protein (TP) (g dL^−1^), total bilirubin (µmol L^−1^), creatinine (mg dL^−1^), urea (mmol L^−1^), uric acid (µmol L^−1^), triglycerides (mmol L^−1^), glucose (mg dL^−1^), cholesterol (mg dL^−1^), sodium (mmol L^−1^), potassium (mmol L^−1^), calcium (mmol L^−1^), magnesium (mmol L^−1^), and iron (µmol L^−1^).

##### 2.2.2.4 Quantification of antioxidant enzymes

Dissected livers were weighed to 1 gram each and homogenized in 5 milliliters of 10 mM phosphate buffer (pH 7.0) at low temperature (4 °C). The homogenized samples were centrifuged at 8000*g* for 10 minutes, and the resulting supernatant was collected in aliquots for enzyme level testing, specifically for superoxide dismutase (SOD), catalase (CAT), and glutathione peroxidase (GPx).

###### 2.2.2.4.1 SOD activity

Measured using a method based on pyrogallol autoxidation. The reaction mixture included 50 mM Tris–EDTA buffer (pH 8.2), 4 mM pyrogallol, and 100 mg liver protein, with absorbance measured at 420 nm for 3 minutes. Enzyme activity was expressed in units per milligram of protein, where one unit represents the enzyme amount required to inhibit pyrogallol auto-oxidation by 50%.

###### 2.2.2.4.2 CAT activity

Determined using the Chance and Maehly method. The reaction mixture contained 200 mg of protein from tissue samples and 10 mM potassium phosphate buffer (pH 7.4), initiated by adding 19.6 mM H_2_O_2_. The decrease in absorbance at 240 nm was observed for 2 minutes. Enzyme activity was expressed in units per milligram of protein, with one unit representing the enzyme amount needed to break down 1.0 millimole of H_2_O_2_ per minute.

###### 2.2.2.4.3 GPx activity

Measured according to the Rotruck method. The reaction mixture included 0.2 M phosphate buffer (pH 7.6), 10 mM sodium azide, 100 mg protein from tissue homogenate, 0.2 mL 1 mM reduced GSH, and 0.1 mL 2 mM H_2_O_2_, with the volume adjusted to 2 mL with deionized water. After a 10 minute incubation at 37 °C, 0.4 mL of 5% TCA was added, followed by centrifugation at 3200*g* for 20 minutes. The supernatant (0.2 mL) was mixed with 1 mL Ellman's reagent and incubated at 20–25 °C for 5 minutes. Absorbance was measured at 412 nm, with enzyme activity expressed in units per milligram of protein, where one unit represents the amount of enzyme needed to consume 1 millimole of GSH per minute.

##### 2.2.2.5 Histopathology

Liver fragments were preserved in a 10% buffered formalin solution to fix their structure and prevent degradation. After fixation, the organs were embedded in paraffin, sectioned, and stained with hematoxylin and eosin (H&E). The stained sections were examined under a light microscope (Axio Lab A1m) to observe liver tissue structure, identify abnormalities or changes, and assess the histological characteristics of the liver.

##### 2.2.2.6 Antioxidant activity: diphenyl-1-picrylhydrazyl (DPPH) method

The radical scavenging activity of the extracts against DPPH free radicals was determined using the method described by, with some modifications. A 1 mL sample solution was combined with methanol DPPH solution (0.1 mM), mixed vigorously, and kept in the dark for 60 minutes. Subsequently, the absorbance of each sample was measured at 517 nm. The scavenging activity was measured as the decrease in absorbance of the samples *versus* the DPPH standard solution. Butylated hydroxytoluene (BHT), trolox, and ascorbic acid were used as positive controls. Results were expressed as the percentage of radical scavenging activity.^28^

#### 2.2.3 Statistical analysis

All data were presented as mean ± standard error of the mean (SEM). Statistical analysis was conducted using one-way analysis of variance (ANOVA). *Post hoc* comparisons were performed using Bonferron and Tukey's multiple comparison tests to analyze the data sets. These analyses were carried out using GraphPad Prism (version 8.0). Statistical significance was considered at *p < 0.05, **p < 0.01, and ***p < 0.001.

### 2.3 Hyperthermia measurements

The heating efficiency was performed using a commercial system, “Nanotherics Magnetherm” with field amplitude and frequency of 170 mT and 332 KHz respectively. Sample absorption rate (SAR) values are calculated by the following equation:1
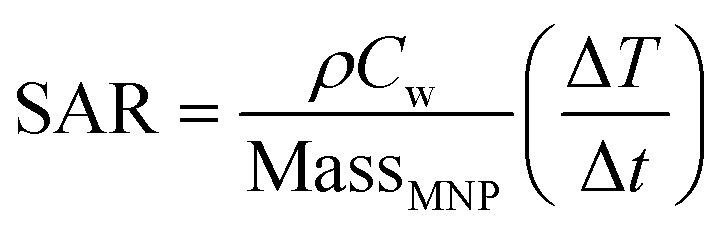
where *ρ* is the colloid density, *C*_w_ is the specific heat of water 
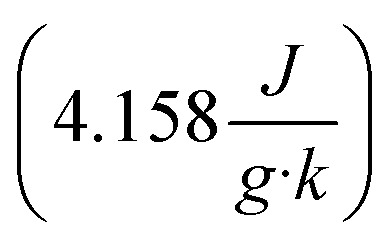
, Mass_MNP_ is the concentration of the magnetic nanoparticles in the suspension, and 
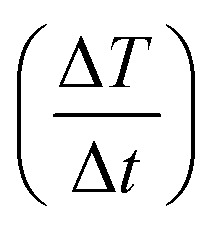
 is the heating rate which represents the initial slope obtained by performing a linear fit of the temperature increase *versus* time.

## 3 Results and discussion

### 3.1 XRD analysis

**Fig. 1:**
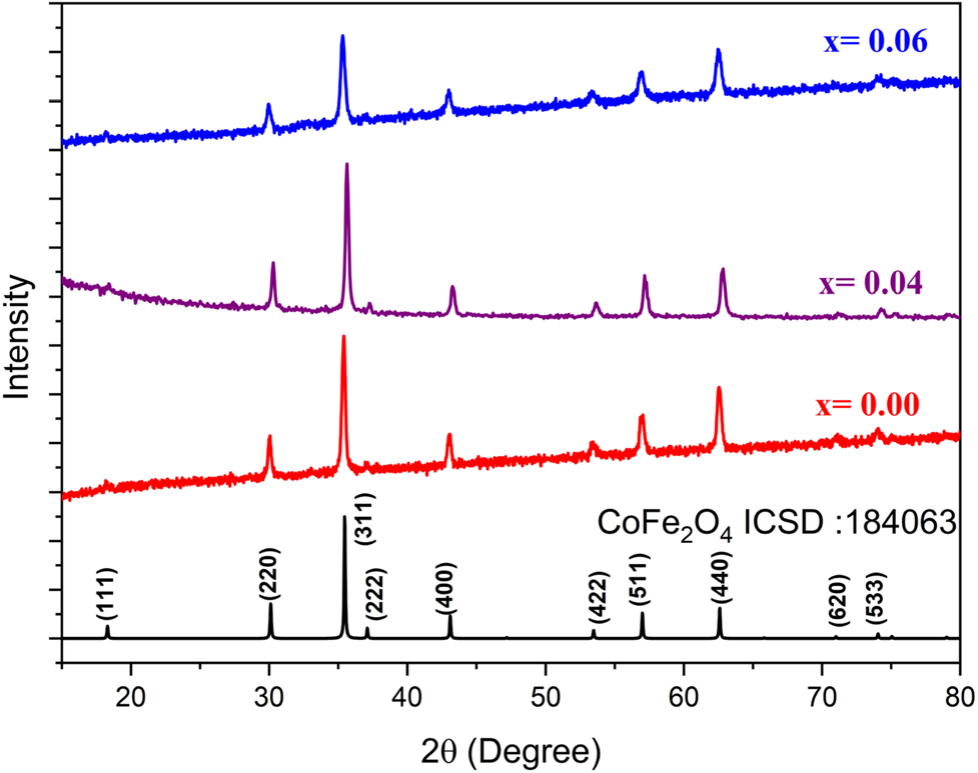
X-ray diffraction patterns of Co_0.5_Mn_0.25_Mg_0.25_Fe_2−*x*_Gd_*x*_O_4_ (*x* = 0.00, 0.04, 0.06) nanoparticles.

Nanoparticles prepared with the composition Co_0.5_Mn_0.25_Mg_0.25_Fe_2−*x*_Gd_*x*_O_4_ (*x* = 0.00, 0.04, 0.06) were examined by X-ray diffraction (XRD) to confirm the nature of the phases formed. The XRD spectra obtained are shown in Fig. 1. Analysis of the spectra reveals that all three samples have a pure phase with a spinel structure. All the observed diffraction peaks are indexed in accordance with the ICDS 184063 reference card of CoFe_2_O_4_ indicating that the crystal structure is well in line with that expected for a spinel. The diffraction spectra show prominent peaks corresponding to the (111), (220), (311), (222), (400), (331), (422), (511), and (440) crystallographic planes located at angles 2*θ* of 30.03°, 35.48°, 37.11°, 43.04°, 53.36°, 56.93°, and 62.53° respectively. For each composition, the spectra show well-defined peaks, confirming the high crystallinity of the samples. Furthermore, no impurities or secondary phases were detected in the XRD spectra, underlining the purity of the synthesized nanoparticles. These results show that the introduction of Gd^3+^ into the spinel structure did not interfere with the formation of the desired phase.

**Fig. 2:**
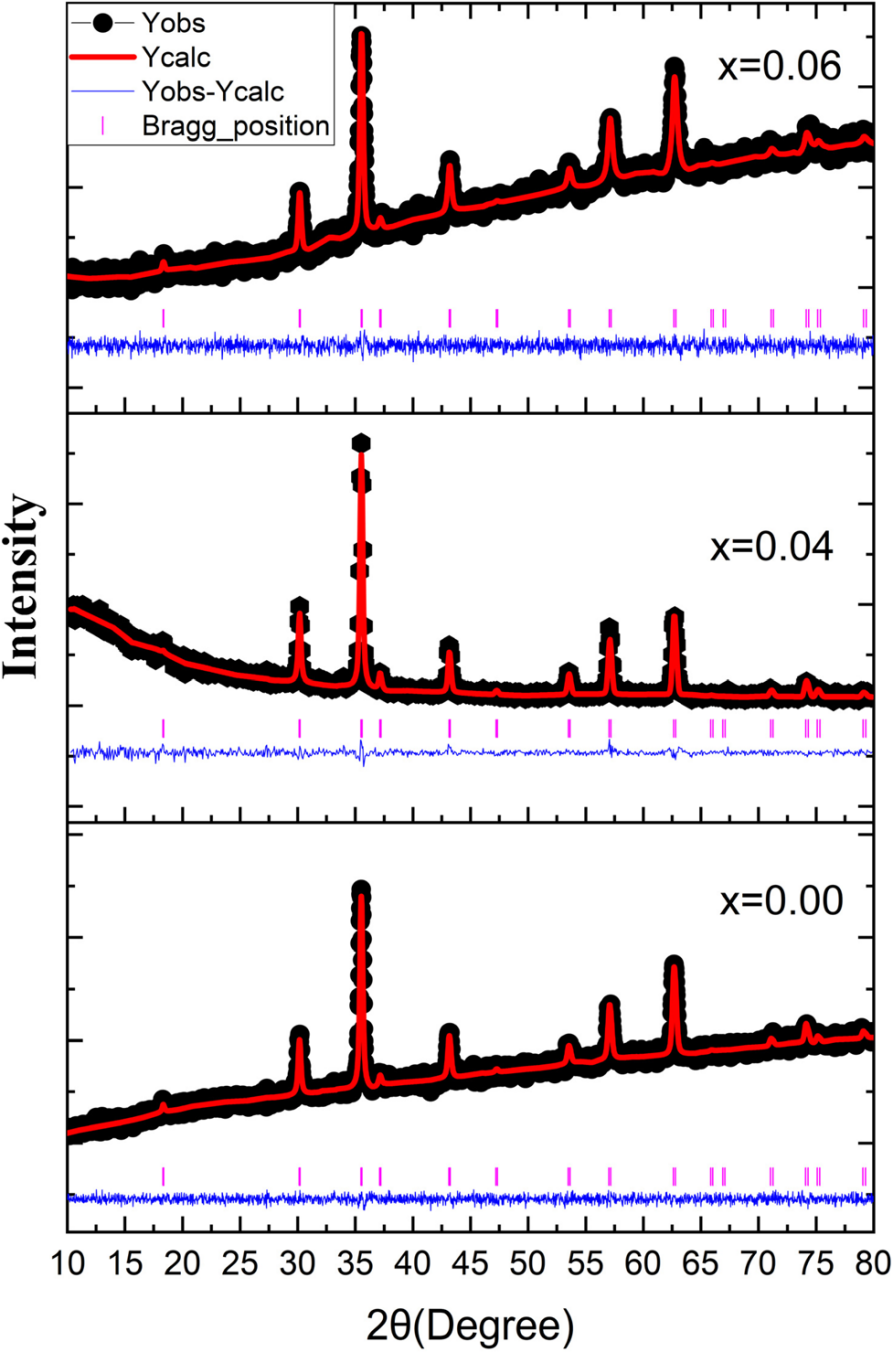
X-ray diffraction and Rietveld refined pattern of Co_0.5_Mn_0.25_Mg_0.25_Fe_2−*x*_Gd_*x*_O_4_ (*x* = 0.00, 0.04, 0.06) nanoparticles.

To obtain more information about the structure of the nanoparticles, the spectra were analysed using the Rietveld refinement method. This analysis enabled the crystal lattice parameters, atomic positions and site occupancy factors to be accurately determined. confirming the integrity and purity of the phases formed. The spectra obtained are shown in Fig. 2, indicating that the nanoparticles are indexed according to the spinel structure of the *Fd*3̄*m* space group. The structural parameters obtained are shown in Table 1.

**Table 1:** Structural parameters, cation distribution, and refinement quality of Gd^3+^ doped Co–Mg–Mn ferrite nanoparticles

Sample	*a* (Å)	*D*_xrd_ (nm)	*d*_x_ (g cm^−3^)	*a*_Th_ (Å)	*r*_A_ (Å)	*r*_B_ (Å)	*u* (Å)	Cation distribution		*χ* ^2^
								A-site	B-site	
*n* = 0.00	8.3819	29.39	5.08	8.4630	0.7575	0.6700	0.3931	Co_0.03_Fe_0.75_Mg_0.08_Mn_0.14_	Co_0.47_Fe_1.25_Mg_0.17_Mn_0.11_	1.095
*n* = 0.04	8.3807	30.75	5.17	8.6429	0.7575	0.7375	0.3931	Co_0.2_Fe_0.65_Mg_0.01_Mn_0.14_	Co_0.3_Fe_1.31_Mg_0.24_Mn_0.11_Gd_0.04_	10.33
*n* = 0.06	8.3772	22.68	5.22	8.4950	0.7575	0.6820	0.3932	Co_0.01_Fe_0.83_Mg_0.08_Mn_0.08_	Co_0.49_Fe_1.11_Mg_0.17_Mn_0.17_Gd_0.06_	1.022
Co_*x*_Fe_(1−*x*−*y*−*z*)_Mg_*y*_Mn_*z*_ Co_(0.5−*x*)_Fe_(1+*x*+*y*+*z*−*n*)_Mg_(0.25−*y*)_Mn_(0.25−*z*)_Gd_*n*_O_4_										

According to the results obtained, the lattice parameter of three samples agrees with the spinel structure parameter.^29,30^ On the other hand, an increase was observed in this parameter for *x* = 0.04 and then decreases for *x* = 0.06. This observation can be explained by the doping effect of Gd^3+^. The increase observed is due to the value of the ionic radius of Gd^3+^, which is comparable with that of Fe^31^ and the decrease after the increase in the doping rate is explained by the strengthening of the super-exchange interactions by Gd^3+^, which leads to a decrease in the bonds between the atoms and. consequently, a decrease in the lattice parameter.^32^

The cationic distribution was determined using the Rietveld method and the results obtained are shown in Table 1. According to the results obtained the three samples present a mixed spinel structure with a distribution of Fe, Co, Mn, and Mg in the two octahedral and tetrahedral sites and Gd in the octahedral site.

Based on the Rietveld refinement, the cation distribution over the tetrahedral (A) and octahedral (B) sites is presented in Table 1. The obtained results confirm that the three compositions adopt a mixed spinel structure, with a non-uniform but systematic redistribution of cations between the two crystallographic sites. For the undoped sample, the A-sites are mainly occupied by Fe^3+^ (0.75) with smaller amounts of Co^2+^, Mg^2+^, and Mn^2+^, while the B-sites accommodate higher concentrations of Co^2+^ (0.47) and Fe^3+^ (1.25). Upon Gd substitution (*x* = 0.04 and 0.06), a noticeable reorganization of the cationic distribution occurs: Co, Mg, and Mn contents gradually increase in the B-site, while Fe tends to slightly decrease in the A-site. Importantly, Gd^3+^ ions due to their larger ionic radius and strong preference for octahedral coordination are exclusively located at the B-sites (0.04 and 0.06, respectively). This selective incorporation of Gd^3+^ leads to local lattice distortions, modifies the A/B cationic ratio, and consequently influences the magnetic interactions governing the overall properties of the spinel structure.

In addition, other characteristic parameters of the spinel structure such as the radii of the octahedral (*r*_A_) and tetrahedral (*r*_B_) sites, the theoretical lattice parameter (*a*_th_) and the internal parameter (*u*) are determined.

The crystallite sizes are determined using the Scherrer relation.^33^ The values found are between (22 and 30 nm) for all three samples. The crystallographic density is found to be between (5.08 and 5.22 g cm^−3^).

### 3.2 FT-IR spectrum

**Fig. 3:**
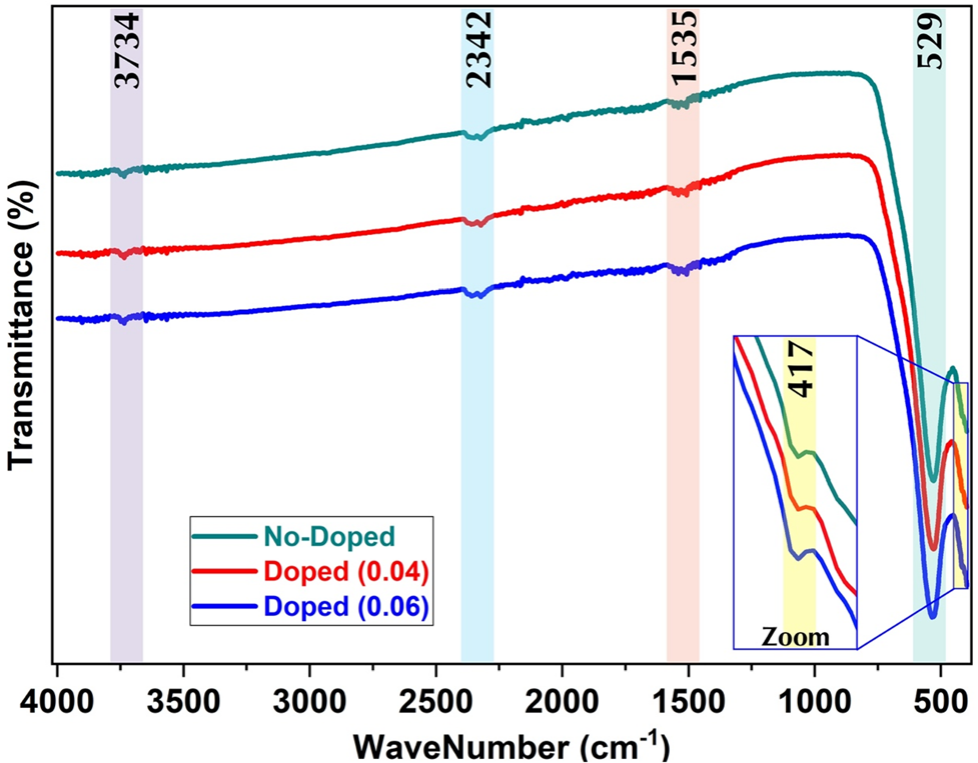
FTIR spectrums of elaborated Mn–Mg–Co ferrite.

Fourier Transform-Infrared spectroscopy (FTIR) was used to confirm the structural results by determining the two main metal–oxygen bands characteristic of the positions of the octahedral and tetrahedral cations and anions.^34–37^ The analysis was performed at wavelengths between 400 and 4000 cm^−1^. It is clear that no significant differences can be noted by comparing all the spectrums (Fig. 3). In all results, the absorption band located at 417 cm^−1^ is attributed to the octahedral metal atoms stretching vibrations M–O. and the second absorption band at 529 cm^−1^ corresponds to the stretching vibration of metal atoms at the tetrahedral site M–O. Moreover, the two absorption bands observed between 400–600 cm^−1^ are the characteristics of the prepared Mn–Mg–Co ferrite because the Mn^2+^, Mg^2+^, Co^2+^, and Fe^3+^ cations can occupy both octahedral and tetrahedral sites, and the Gd^3+^ cation can occupy the octahedral sites.^34–39^ In addition, the FTIR spectrums (Fig. 3) show three very small absorption bands located above 1500 cm^−1^. The band at 1535 cm^−1^ corresponds to H–O–H bending vibration; the bands at 2342 cm^−1^ and 3734 cm^−1^ are assigned to symmetric and antisymmetric O–H stretching as a consequence of the adsorbed water [6.7].

### 3.3 X-ray photoelectron spectroscopy investigations

**Fig. 4:**
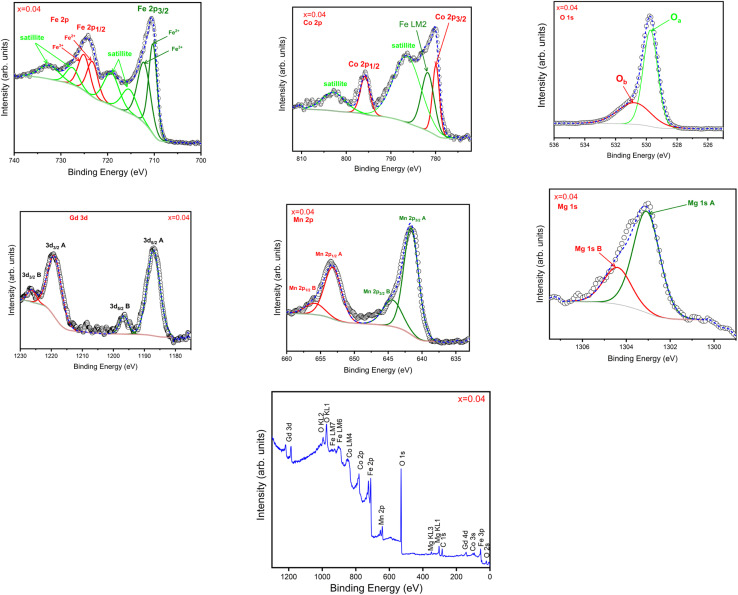
Deconvoluted XPS spectra of Co_0.5_Mn_0.25_Mg_0.25_Fe_2−*x*_Gd_*x*_O_4_ nanoparticles for *x* = 0.04.

X-ray photoelectron spectroscopy (XPS) analysis was used to characterize Co_0.5_Mn_0.25_Mg_0.25_Fe_2−*x*_Gd_*x*_O_4_ nanoparticles. This technique makes it possible to determine the surface elemental composition as well as the oxidation states of the elements present. The results obtained provide crucial information about the valence states of cobalt and iron and the doping elements, which is essential for understanding the chemical and magnetic properties of nanoparticles. Interpretation of the XPS spectra will make it possible to identify characteristic peaks and assess variations in oxidation states, thereby contributing to a better understanding of the structure and properties of nanoparticles. Fig. 4 shows the spectrum of Co_0.5_Mn_0.25_Mg_0.25_Fe_2−*x*_Gd_*x*_O_4_ nanoparticles (*x* = 0.04). The XPS core spectra of Co 2p exhibits four distinct peaks. The first peak can be deconvoluted into two Gaussian peaks: the first, situated at 780 eV, corresponds to Co 2p_3/2_, while the second, observed at 782.0 eV, is attributed to the Fe LM2 Auger peak, overlapping with the Co 2p_3/2_ peak.^41^ Each of the other three peaks can be fitted with a single Gaussian envelope. The 2p_1/2_ peak appears at about 795.6 eV, additionally, two shake-up satellite peaks are observed at 786.5 eV and 7802.4 eV, 8 eV, respectively. The Co 2p_3/2_ and 2p_1/2_ peaks exhibit a spin–orbit splitting energy (Δ*S*) of 15.6 eV. The observed positions of these peaks align closely with standard values for the Co^2+^ state, indicating the absence of Co clusters, these values are in good agreement with those previously reported in the literature.^40,42,43^ The XPS core spectra of Fe 2p displays two main peaks. The first peak (2p_3/2_) can be resolved into three Gaussian peaks situated at 710.5 eV and 712.7 eV, corresponding to Fe^2+^ and Fe^3+^, respectively, along with a satellite peak at 715.5 eV. An additional satellite peak is detected at 719.4 eV. The second peak (2p_1/2_) is similarly deconvoluted into three Gaussian peaks centered at 723.6 eV and 725.5 eV, representing Fe^2+^ and Fe^3+^, respectively, and featuring a satellite peak at 727.8 eV. Another satellite peak is located at 732.8 eV. Similar spectra have been reported in doped SrM and BaM ferrites.^41,44^ The O 1s spectrum is deconvoluted into two peaks at 529.7 eV (*O*_a_) and 530.7 eV (*O*_b_). The first peak is ascribed to O^2−^ ions within the lattice, while the second peak is assigned to oxygen vacancies – oxygen ions surrounded by vacant oxygen sites within the lattice.^45,46^ The core level spectrum of Gd 4d is illustrated in the figure. This spectrum displays two prominent peaks located at 141.8 eV and 147.4 eV, corresponding to Gd^3+^ 4d_5/2_ and Gd 4d_3/2_, respectively. The positions of these peaks, along with the observed spin–orbit splitting energy, prove the presence of the Gd^3+^ valence state in the samples.^47,48^ The XPS spectra of Mn 2p can be resolved into four peaks at 641.6 eV, 644.5 eV, 653.2 eV, and 655.8 eV, corresponding to Mn^3+^ (2p_3/2_), Mn^4+^ (2p_3/2_), Mn^3+^ (2p_1/2_), and Mn^4+^ (2p_1/2_) species, respectively.^49,50^ The core level spectrum of Mg 1s is shown in Fig. 4, the spectrum is deconvoluted into two Gaussian peaks located at 1303.4 eV and 1304.6 eV, attributed to Mg^2+^ and Mg^3+^, respectively.^51^

The incorporation of Gd^3+^ into Co_0.5_Mn_0.25_Mg_0.25_Fe_2−*x*_Gd_*x*_O_4_ nanoparticles preserves the spinel structure, as evidenced by the FTIR spectra showing characteristic bands at 417 cm^−1^ and 529 cm^−1^, corresponding to the stretching vibrations of cations at octahedral and tetrahedral sites, with Gd^3+^ preferentially occupying the octahedral sites. XPS analysis further confirms the presence of Fe^2+^/Fe^3+^ and Co^2+^ and demonstrates the effective incorporation of Gd^3+^ on octahedral sites, while Mn and Mg are distributed across both octahedral and tetrahedral sites. The O 1s spectra reveal the coexistence of O^2−^ ions and oxygen vacancies, indicating that Gd^3+^ doping slightly modifies the local Fe–O environment without disrupting the overall structure. These combined observations suggest that Gd^3+^ stabilizes the octahedral Fe–O coordination, potentially enhancing Fe^3+^–O–Fe^3+^ superexchange interactions and favorably influencing the chemical and magnetic properties of the doped ferrites.

### 3.4 Morphological study

**Fig. 5:**
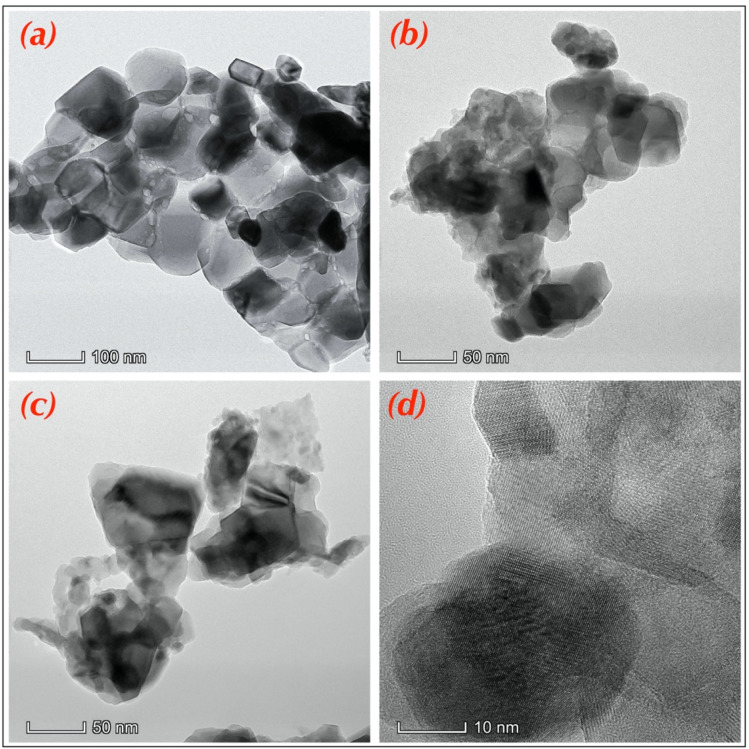
TEM images of elaborated Mn–Mg–Co ferrite: (a) no-doped. (b) Doped 0.04 and (c and d) doped 0.06.

The morphology of the samples was investigated using Transmission Electron Microscopy (TEM). The TEM images are summarized in Fig. 5. As can be seen in the images, the nanoparticles are not dispersed, and some have agglomeration. which is observed in all samples due to the magnetic nature of the materials.^36,37,52^ In addition, the nanoparticles showed an irregular shape, and the same spherical or cubic nanoparticles were observed (Fig. 5). It is clear that all nanoparticles have nanometric size, as confirmed by the results obtained from the statistical values obtained by ImageJ Software. The three histograms generated from the results are shown in Fig. 5 where they show the size distribution range of nanoparticles, and the average size for each sample. Indeed, according to the literature, many parameters can strongly influence nanoparticle sizes and morphology, such as experimental conditions, synthesis methods, and doping elements.^53–55^

**Fig. 6:**
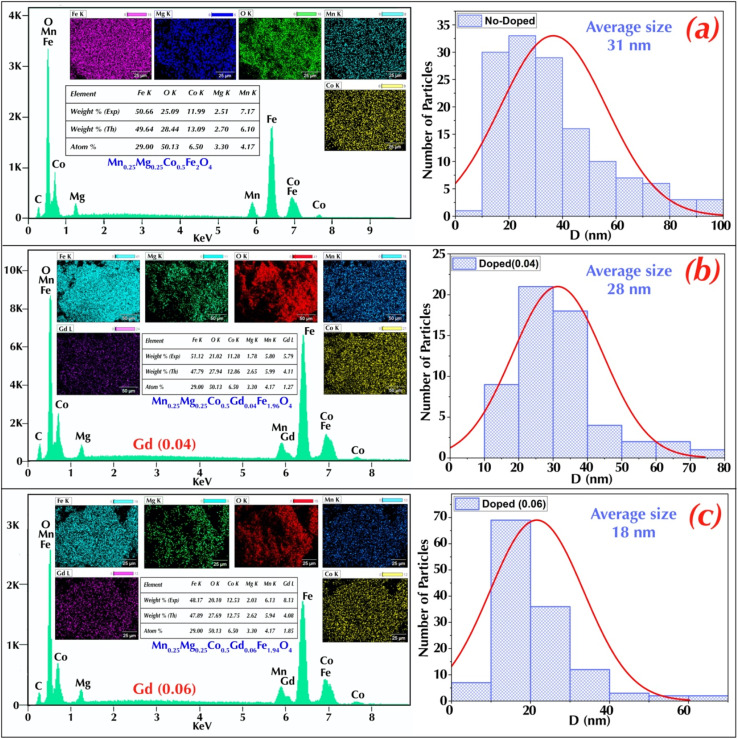
Size distribution. EDS and mapping analysis of elaborated Mn–Mg–Co ferrite: (a) no-doped. (b) Doped 0.04 and (c) doped 0.06.

To inspect the chemical composition and the elemental distribution in the synthesized samples, the third investigation was made by the Energy-Dispersive Detector (EDS). The EDS spectrums and elemental mapping are shown in Fig. 6. All peaks corresponding to Fe, Mn, Mg, Co, Gd, and O are present in the EDS spectrums without any peaks of impurities. There is confirmed by each mapping picture, also shows the uniform distribution of all the elements of each sample. The small peak in C is related to the carrier of the equipment. And for each element, the experimental and theoretical composition percentages (calculated by eqn (2)) agree with one another, as presented in the tables (Fig. 6).2
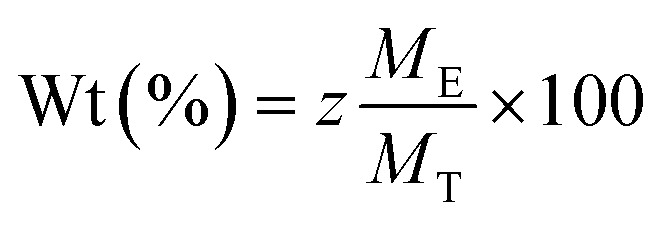
where *M*_T_ is the total molar mass of the sample. *M*_E_ is the molar mass of the element, and *z* refers to the number of elements.

### 3.5 Magnetic properties

**Fig. 7:**
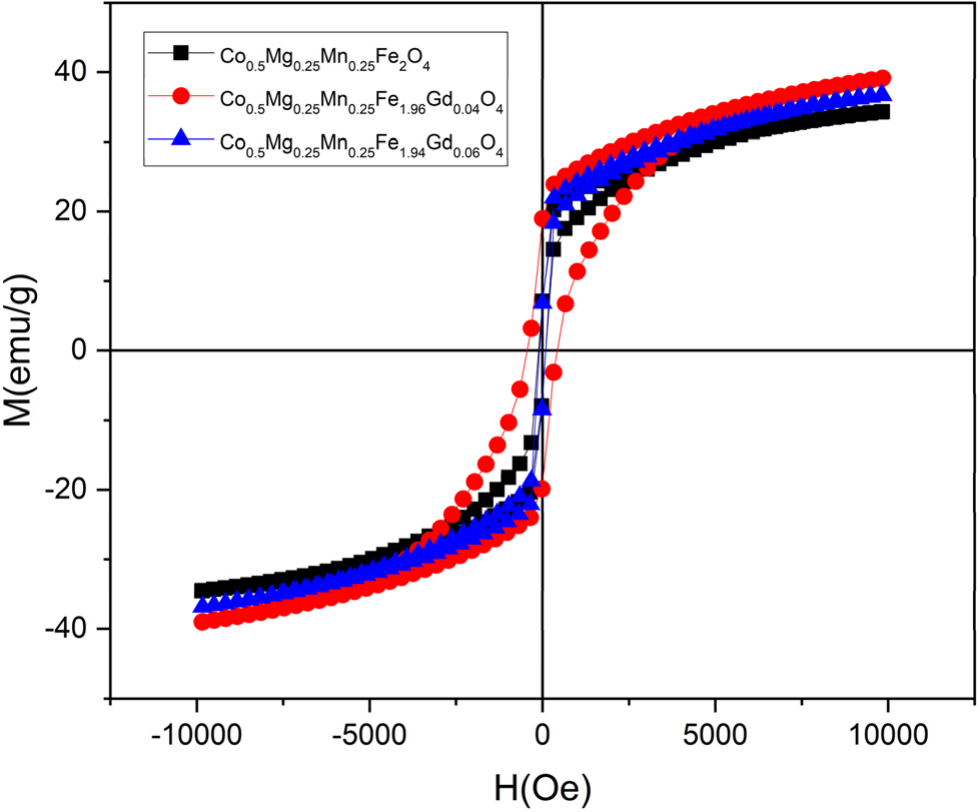
Magnetization (*M*) *versus* applied magnetic field (*H*) curves at room temperature for Co_0.5_Mn_0.25_Mg_0.25_Fe_2−*x*_Gd_*x*_O_4_ nanoparticles with different Gd^3+^ concentrations (*x* = 0.00, 0.04, 0.06).

The magnetic properties of the synthesised nanoparticles Co_0.5_Mn_0.25_Mg_0.25_Fe_2−*x*_Gd_*x*_O_4_ (*x* = 0.00; 0.04; 0.06) were examined using a VSM at a temperature of 300 K. The hysteresis cycles obtained are shown in Fig. 7. Magnetic parameters such as saturation magnetisation (*M*_s_), remanent magnetisation (*M*_r_) and coercive field (*H*_c_) are summarised in the table. From these cycles, it can be seen that the nanoparticles exhibit ferrimagnetic behaviour.

The saturation magnetisation (*M*_s_) is measured for *x* = 0.00. *M*_s_ is 39.00 emu g^−1^. As the Gd concentration increases to *x* = 0.04. *M*_s_ reaches 45.69 emu g^−1^. indicating an improvement in the maximum magnetisation possible. This may be due to a better alignment of the magnetic moments in the material due to the addition of Gd.^56^ This increase can also be attributed to the substitution of Gd^3+^ in place of Fe^3+^. which can enhance ferrimagnetic super-exchange interactions between ions in the crystal lattice.^57^ According to the literature, the introduction of rare earths such as Gd^3+^ can increase the density of magnetic moments per ion and improve the overall magnetic properties of ferrites. The magnetic moment of Fe^3+^ is about 5 µ_B_ (bohr magnetions). while that of Gd^3+^ is about 7.94 µ_B_. The increase in *M*_s_ at *x* = 0.04 could therefore be due to the higher magnetic contribution of Gd^3+^ compared to Fe^3+.^^58–60^

To determine the effect of the cation distribution on the saturation magnetisation, the Néel model was used to calculate the theoretical magnetic moment of the samples according to the following eqn (3):

3 *M*_Cal_ = *M*_B_ − *M*_A_

where *M*_B_ is the magnetic moment at the octahedral site and *M*_A_ is the magnetic moment at the tetrahedral site.

The experimental magnetic moment was calculated using the following eqn (4):4
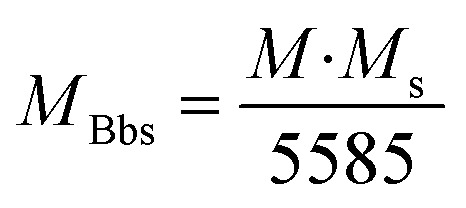
where *M* is the molecular weight of the samples and *M*_s_ is the saturation magnetisation.

The values of the calculated and observed moments are shown in the Table 2. A significant difference was observed between the measured and calculated moments. This discrepancy can be explained by the spin–orbit coupling effect, which influences the distribution of spin and orbit magnetic moments in the material, as well as by the presence of cations with large spin moments. In addition, the spin canting effect, which results from a misalignment of the spin moments in a material, can also contribute to this difference. This interaction can modify the values of the observed moments by introducing a magnetic anisotropy that is not always fully integrated into theoretical models. Nanoparticle confinement effects and cation distribution can exacerbate these differences, as spin–orbit coupling, cation spin moments, and spin canting can interact in complex ways with other magnetic effects, such as exchange interactions. Consequently, discrepancies between measured and calculated values may reflect aspects not taken into account in the theoretical calculations.^61,62^

**Table 2:** Magnetic parameters of Co_0.5_Mn_0.25_Mg_0.25_Fe_2−*x*_Gd_*x*_O_4_ nanoparticles (*x* = 0.00, 0.04, 0.06) derived from VSM measurements

	*H*_c_ (Oe)	*M*_s_ (emu g^−1^)	*M*_r_ (emu g^−1^)	*M*_r_/*M*_s_	*n*_B_ (obs) (µ_B_)	*n*_B_ (cal) (µ_B_)	*θ*_Y–K_ (°)
*x* = 0.00	108	39.00	7.28	0.18667	1.57089346	3.89	59.78
*x* = 0.04	427	45.69	18.84	0.41234	1.87357633	3.81	53.45
*x* = 0.06	96	42.37	6.85	0.16167	1.75285866	4.00	56.87

The existence of spin canting in the samples can be evident by determination of the Yafet–Kittle angle. denoted as *θ*_Y–K_. The angle *θ*_Y–K_ is calculated using the formula (5);^63^

5 *n*_exp_ = *M*_B_ cos (*θ*_Y–K_) − *M*_A_

where *M*_B_ and *M*_A_ are the magnetic moments of sites B and A. respectively and *n*_exp_ is the experimental magnetic moment.

The calculated Yafet–Kittle angles *θ*_Y–K_ are presented in the Table 2 the three samples have angles of the same order of magnitude, confirming the presence of spin canting.^64^

On the other hand, the saturation magnetization (*M*_s_) of Co_0.5_Mn_0.25_Mg_0.25_Fe_2−*x*_Gd_*x*_O_4_ ferrites is closely dependent on the distribution of ions between the tetrahedral (A) and octahedral (B) sites. For *x* = 0.00, the A sites are strongly dominated by Fe^3+^ (0.75), while the B sites contain a high proportion of Fe^3+^ (1.25) and Co^2+^ (0.47). This distribution creates a moderate difference between the magnetic moments of the A and B sublattices, resulting in an *M*_s_ of 39 emu g^−1^. When *x* increases to 0.04, partial migration of Co^2+^ and Fe^3+^ to the A sites, combined with the introduction of Gd^3+^ into the B sites (0.04), enhances the A–B superexchange interactions and reduces spin canting, as indicated by the decrease in the Yafet–Kittel angle (53.45°). This redistribution makes the moments of the two sublattices more antiparallel and better aligned, explaining the significant increase in *M*_s_ to 45.69 emu g^−1^. In contrast, for *x* = 0.06, the B sites become saturated with non-magnetic or weakly magnetic ions (Mg^2+^, Mn^2+^) and Gd^3+^, which increases cationic disorder and promotes greater canting (*θ* = 56.87°). This disruption of magnetic order reduces the efficiency of the A–B coupling and leads to a slight decrease in *M*_s_ (42.37 emu g^−1^).^65–67^

To better understand the exchange interactions, present in the samples, we calculated the interatomic distances using the equations cited in the literature.^56^ The values found are shown in the Tables 3 and 4.

**Table 3:** The calculated inter-ionic distances between cation–anion (Me–O) and cation–cation (Me–Me) of samples Co_0.5_Mn_0.25_Mg_0.25_Fe_2−*x*_Gd_*x*_O_4_ (*x* = 0.00; 0.04; 0.06)

Parameters	*b* (Å)	*c* (Å)	*d* (Å)	*e* (Å)	*f* (Å)	*p* (Å)	*q* (Å)	*r* (Å)	*s* (Å)
*x* = 0.00	2.963449	3.474952	3.629469	5.444204	5.132845	1.943763	2.077508	3.978125	3.71706
*x* = 0.04	2.963025	3.474455	3.62895	5.443424	5.13211	1.943484	2.077211	3.977556	3.716528
*x* = 0.06	2.961787	3.473004	3.627434	5.441151	5.129966	1.941835	2.077794	3.978673	3.71546

**Table 4:** The calculated bond angle of samples Co_0.5_Mn_0.25_Mg_0.25_Fe_2−*x*_Gd_*x*_O_4_ (*x* = 0.00; 0.04; 0.06)

Parameters	*θ*_1_ (°)	*θ*_2_ (°)	*θ*_3_ (°)	*θ*_4_ (°)	*θ*_5_ (°)
*x* = 0.00	119.5329907	130.4605783	99.3342499	127.2736324	65.06522108
*x* = 0.04	119.5329907	130.4605783	99.3342499	127.2736324	65.06522108
*x* = 0.06	119.5016889	130.3557516	99.39249196	127.2850715	64.99489585

The relationship between interatomic distances and exchange interactions for engineered nanoparticles is evident from the variations observed. For *x* = 0.00, the saturation magnetisation (*M*_s_) is 39.00 emu g^−1^. By increasing *x* to 0.04, the interatomic distances decrease slightly, and the angles change, leading to an increase in *M*_s_ to 45.69 emu g^−1^. This increase can be attributed to a strengthening of the super-exchange interactions (A–O–B) between the metal ions *via* the oxygen ions, due to the contraction of the crystal structure.^68^ However, for *x* = 0.06, although the interatomic distances continue to decrease and the angles still vary, *M*_s_ decreases slightly to 42.37 emu g^−1^. This decrease could be due to an excess of Gd^3+^ disrupting the optimal exchange interactions.^69^ Thus, variations in interatomic distances and angles directly influence the exchange interactions, significantly affecting the saturation magnetisation in these doped cobalt ferrites.

The values of the corrective fields were determined and are shown in the Table 2. At a concentration of 4% Gd^3+^(*x* = 0.04), the coercivity of the nanoparticles increases significantly at 427 Oe, mainly due to the enhancement of the magnetocrystalline anisotropy induced by Gd^3+^. Gd^3+^increases the anisotropic fields, making magnetisation more difficult to reverse.^70^ At this concentration, the size of the nanoparticles is 30.75 nm, which favours a higher coercivity. However, at a higher concentration of 6% Gd^3+^(*x* = 0.06), the coercivity decreases to 96 Oe, despite the presence of Gd^3+^. This decrease is due to a reduction in the size of the nanoparticles to 22.68 nm, which leads to an increase in surface effects and a disruption of internal magnetic interactions.^71^ Smaller nanoparticles can also introduce structural defects or complex anisotropies, reducing the effectiveness of beneficial magnetocrystalline anisotropy and leading to a decrease in coercivity. Thus, variations in nanoparticle size and Gd^3+^ concentrations influence the coercivity of the material in complex ways, with larger nanoparticle sizes and optimal Gd^3+^ concentrations favoring higher coercivity.^72,73^

The ratio (*M*_r_/*M*_s_) was calculated for each sample, as shown in the table. Compared with the undoped sample (*x* = 0.00), the ratio (*M*_r_/*M*_s_) increases for the sample with *x* = 0.04, reaching 0.41234. Then, the ratio decreases for the sample with *x* = 0.06, returning to 0.16167. Then, the ratio decreases for the sample with *x* = 0.06, returning to 0.16167 A ratio (*M*_r_/*M*_s_) greater than 0.5 indicates the presence of a single magnetic domain in the nanoparticles, while a ratio less than 0.5 suggests the presence of several magnetic domains.^74,75^ For all the samples prepared, the values obtained are less than 0.5, which confirms the presence of several magnetic domains in these nanoparticles. This observation is important because it improves our understanding of the magnetic behaviour of these nanoparticles and opens up new prospects for their use in various technological applications.

### 3.6 Magnetic hyperthermia analysis

Magnetic fluid hyperthermia (MFH) is a cancer treatment method, which uses the heat dissipated by magnetic nanoparticles (MNPs) under an alternating magnetic field (AMF) to kill cancerous cells.^76–79^ The specific absorption rate (SAR) is the amount of heat released by a unit gram of the magnetic material in unit time under AMF.^4^ SAR values of MNPs are affected by many parameters, such as size, structure, saturation, coercivity, remanence, field amplitude and frequency of AMF.^76^

**Fig. 8:**
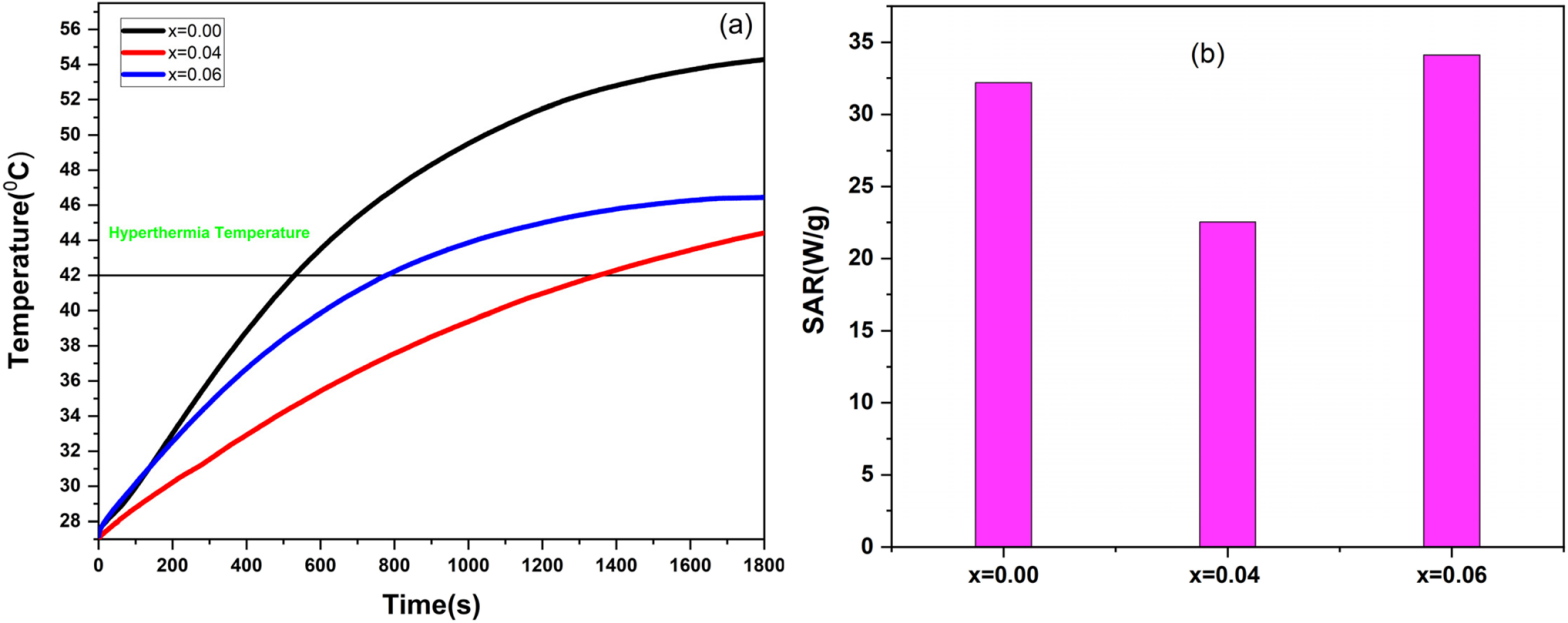
(a) Temperature rise at frequency *f* = 332 KHz and field amplitude of 170 mT and (b) SARs values.

Fig. 8a shows the temperature rise under an AMF of Co_0.5_Mn_0.25_Mg_0.25_Fe_2−*x*_Gd_*x*_O_4_ (*x* = 0.00, 0.04, 0.06) dispersed in deionized water, and Table 5 summarizes the parameters deduced from the heating ability. All the NPs showed a very good heating ability and reached magnetic hyperthermia temperature in a relatively short time. For instance, 42 °C is reached for the sample with Gd (0%) in 8 minutes, while the sample with Gd (6%) took around 22 minutes to reach the same temperature (Table 5).

**Table 5:** Parameters deduced from temperature rise for at *f* = 332 kHz and field amplitude *H*_0_ = 170 Oe

Gd (%)	Concentration (mg mL^−1^)	Maximum temperature (°C)	The time needed to reach hyperthermia temperature 42 °C (minutes)	SAR (W g^−1^) 2–15 s
0	5	54.28	8	33
4	5	46.45	13	34.12
6	5	44.42	22	22.53

The values of SAR were founded to be equal to 33, 34.12, and 22.53 Wg^−1^ for 0%, 4%, and 6% of Gd respectively (Fig. 8b), indicating the effect of Gd^3+^ on SAR values, revealing a notable decrease in thermal efficiency for the highest Gd^3+^ content. These results confirm that Gd^3+^ incorporation modifies the magnetic and thermal properties, likely by affecting the crystal structure, saturation magnetization, and coercivity of the particles. Compared with the literature, our SAR values fall within a moderate range but remain sufficient for magnetic hyperthermia applications. Reported data show a wide dispersion of performances depending on nanoparticle composition, morphology, and surface modifications. For instance, extremely high SAR values have been obtained for Fe_3_O_4_/graphene oxide nanohybrids (5020 W g^−1^),^80^ Zn-doped magnetite (600 W g^−1^),^81^ and folic-acid-functionalized magnetite (530 W g^−1^).^82^ Intermediate SAR values have been reported for MnFe_2_O_4_ (217.62 W g^−1^)^83^ and silica-encapsulated magnetite (190 W g^−1^).^84^ In contrast, the lowest performances were observed for NiFe_2_O_4_ (11 W g^−1^)^85^ and Zn–Mn co-doped magnetite (37.7 W g^−1^).^86^ Therefore, although our nanoparticles exhibit lower SAR values than highly optimized high-performance systems, they are comparable to certain experimentally tested materials, while maintaining a therapeutic temperature rise time compatible with biomedical applications.^17,87^

### 3.7 *In vivo* investigation of hepatoprotective effects

#### 3.7.1 Analysis of antioxidant enzyme levels

**Fig. 9:**
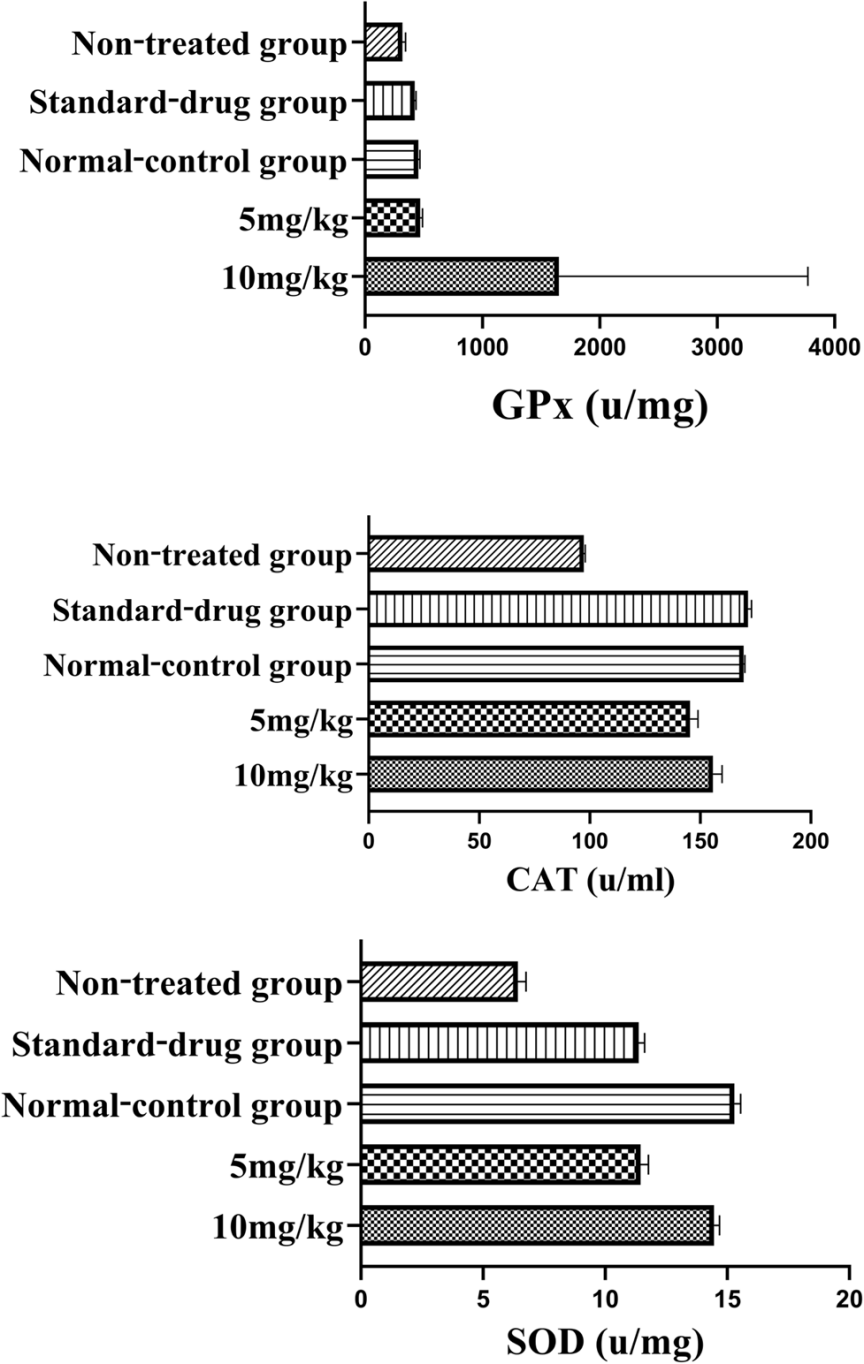
Analysis of antioxidant enzyme levels (GPx, CAT, and SOD).

The analysis of antioxidant enzyme levels (GPx, CAT, and SOD) highlights the potential hepatoprotective effects of Co_0.5_Mn_0.25_Mg_0.25_Fe_2−*x*_Gd_*x*_O_4_ (*x* = 0.04) nanoparticles Fig. 9. For GPx, there were no significant differences between the various groups (*p* > 0.05 for all comparisons), suggesting that nanoparticle treatments do not significantly affect the activity of this enzyme under the tested conditions. However, the levels of CAT and SOD provide more revealing insights into the hepatoprotective effects of the nanoparticles. For CAT, treatments at 10 mg kg^−1^ and 5 mg kg^−1^ showed significant differences compared to the ethanol-induced liver injury group, with *p*-values of 0.0073 and 0.0081, respectively. This indicates that the nanoparticles enhance catalase activity in this liver stress condition. Additionally, the 10 mg kg^−1^ treatment showed a significant difference compared to the standard drug (silymarin), with a *p*-value of 0.0212. These results suggest that the nanoparticles increase catalase activity, a key enzyme in detoxifying peroxides, thereby reducing oxidative stress and cellular damage in the liver. Regarding SOD, nanoparticles significantly increased the levels of this enzyme compared to the ethanol-induced liver injury group (*p* = 0.0021 for 10 mg kg^−1^ and *p* < 0.0001 for 5 mg kg^−1^), highlighting a notable improvement in the liver's ability to neutralize free radicals. The 10 mg kg^−1^ treatment also showed a significant difference compared to the standard drug (*p* = 0.0110). These results indicate that nanoparticles enhance the liver's antioxidant capacity, which is crucial for protecting against oxidative damage.

Our findings confirm the hepatoprotective role of nanoparticles against oxidative stress, in agreement with previous reports. Analysis of antioxidant enzyme levels (GPx, CAT, and SOD) revealed that, while no significant changes were observed for GPx (*p* > 0.05), both CAT and SOD activities were significantly increased by nanoparticle treatments, particularly at 10 mg kg^−1^ and 5 mg kg^−1^, compared to the ethanol-induced liver injury group. These enhancements reflect an improved ability to detoxify peroxides (CAT) and neutralize free radicals (SOD), two key mechanisms in the prevention of oxidative damage. The 10 mg kg^−1^ treatment produced a greater effect than the standard drug silymarin for both enzymes, highlighting a marked hepatoprotective potential. These results are consistent with the work of Salih *et al.*^88^ and Cherian *et al.*,^89^ who also reported a notable stimulation of antioxidant activity (CAT, GSH, GSH-Px or DPPH) following treatments with functionalized or green-synthesized ferrites. In contrast, certain formulations such as Mn/Ce ferrites or high-dose NiFe_2_O_4_ have been associated with a decrease in *in vivo* antioxidant defenses,^90,91^ indicating that composition, dosage, and surface functionalization largely determine the bioactive profile of ferrite nanoparticles.

#### 3.7.2 Evaluation of antibacterial activity: MIC and MBC analysis of nanoparticles

**Fig. 10:**
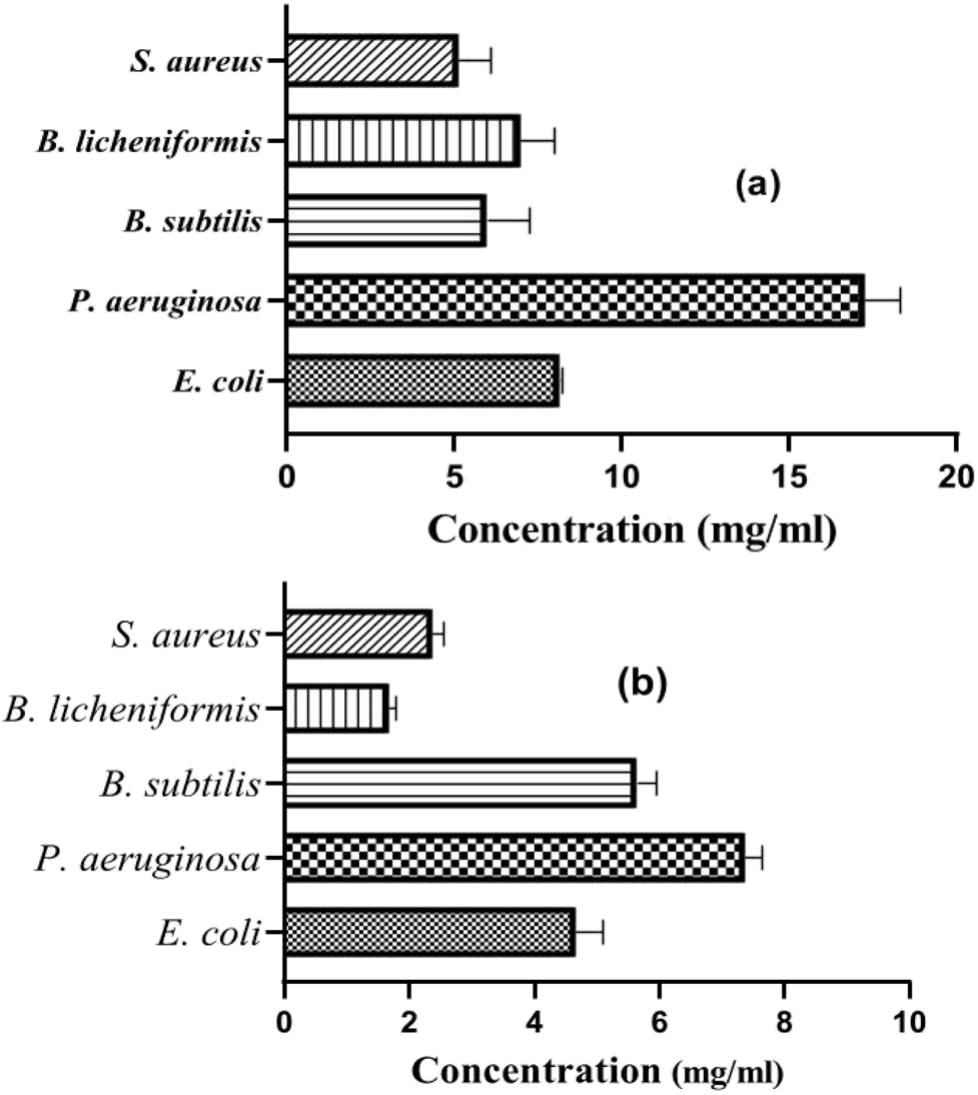
Evaluation of MBC (a) and MIC (b) of nanoparticles on Gram-negative and Gram-positive bacterial strains.

Gram-negative bacteria, *E. coli* and *P. aeruginosa*, exhibit higher average MBC values (8.16 µg mL^−1^ and 17.28 µg mL^−1^, respectively) and MIC values (4.66 µg mL^−1^ and 7.37 µg mL^−1^, respectively) compared to Gram-positive bacteria, indicating a lower sensitivity to Co_0.5_Mn_0.25_Mg_0.25_Fe_2−*x*_Gd_*x*_O_4_ (*x* = 0.04) nanoparticles. *P. aeruginosa* shows the highest resistance among the tested strains, requiring substantial concentrations to achieve effective bactericidal and inhibitory activity (Fig. 10).

Conversely, Gram-positive bacteria such as *B. subtilis*, *B. licheniformis*, and *S. aureus* display lower MBC and MIC values, demonstrating increased sensitivity to nanoparticles. Among these, *S. aureus* is the most sensitive with an average MBC of 5.12 µg mL^−1^ and an average MIC of 2.37 µg mL^−1^. The extremely low MIC values for *B. licheniformis* (1.67 µg mL^−1^) suggest a particularly high efficacy of Co_0.5_Mn_0.25_Mg_0.25_Fe_2−*x*_Gd_*x*_O_4_ (*x* = 0.04) nanoparticles in inhibiting this bacterium.

The comparative analysis shows that bacterial sensitivity to Co_0.5_Mn_0.25_Mg_0.25_Fe_2−*x*_Gd_*x*_O_4_ (*x* = 0.04) nanoparticles varies markedly depending on whether the strain is Gram-positive or Gram-negative, which is consistent with observations in the literature. In our study, Gram-negative bacteria (*Escherichia coli* and *Pseudomonas aeruginosa*) exhibited higher average MBC values (8.16 µg mL^−1^ and 17.28 µg mL^−1^, respectively) as well as higher MIC values (4.66 µg mL^−1^ and 7.37 µg mL^−1^, respectively), indicating greater tolerance to nanoparticles. *P. aeruginosa* emerged as the most resistant strain, requiring substantially higher concentrations to achieve bactericidal and inhibitory effects, which can be attributed to the low permeability of its outer membrane rich in lipopolysaccharides and the efficiency of its efflux pump systems.^92^ Conversely, Gram-positive bacteria (*Bacillus subtilis*, *Bacillus licheniformis*, *Staphylococcus aureus*) displayed significantly lower MBC and MIC values, reflecting higher susceptibility to nanoparticles. *S. aureus* stood out with an average MBC of 5.12 µg mL^−1^ and an average MIC of 2.37 µg mL^−1^, while *B. licheniformis* showed the lowest MIC (1.67 µg mL^−1^), suggesting a particularly effective interaction between the nanoparticle surface and the bacterial cell wall.

These findings are consistent with recent studies on doped ferrites and nanocomposites. For instance, Khalid *et al.*^93^ demonstrated that integrating CoTi_0.2_Fe_1.8_O_4_ into a graphene oxide (GO) matrix drastically reduced MIC and MBC values against *P. aeruginosa* (0.046 and 0.093 mg mL^−1^, respectively) due to enhanced charge separation and increased generation of reactive oxygen species (ROS). Dabagh *et al.*^94^ reported that CuFe_2_O_4_ exhibited remarkable activity against *E. coli* (MIC/MBC = 400/800 µg mL^−1^), outperforming ZnFe_2_O_4_ and MnFe_2_O_4_, confirming the importance of the metal cation type. Similarly, Sharma *et al.*^95^ found that silver doping in MgFe_2_O_4_ significantly enhanced antibacterial activity, particularly against *S. aureus*, due to Ag release and increased active surface area. Aisida *et al.*^92^ observed that Mg doping in CoFe_2_O_4_ improved *E. coli* inhibition compared to Mn doping, while Ghanbari *et al.*^96^ showed that Cr substitution in CuFe_2_O_4_ reduced crystallite size and enhanced bactericidal efficiency. Overall, our results confirm that the higher resistance of Gram-negative strains is a key factor to consider in designing antibacterial nanoparticles, and that targeted optimization of chemical composition (dopants, crystallite size, specific surface area) or the use of conductive supports (GO) can significantly lower MIC and MBC values, especially for the most resistant strains such as *P. aeruginosa*.^97^

#### 3.7.3 Serum biochemical markers

The statistical analysis revealed highly significant differences (*P* < 0.0001). *Post hoc* comparisons using Tukey's and Bonferroni's tests consistently showed that animals in the untreated group exhibited the most unfavorable biological profiles. In contrast, those that received either the reference drug or the tested doses (5 and 10 mg kg^−1^) of Co_0.5_Mn_0.25_Mg_0.25_Fe_2−*x*_Gd_*x*_O_4_ (*x* = 0.04) nanoparticles displayed improved outcomes. Overall, untreated animals had markedly higher values across the main parameters compared with all treated groups (*P* < 0.001), underscoring the protective effect of the nanoparticle treatments.

**Fig. 11:**
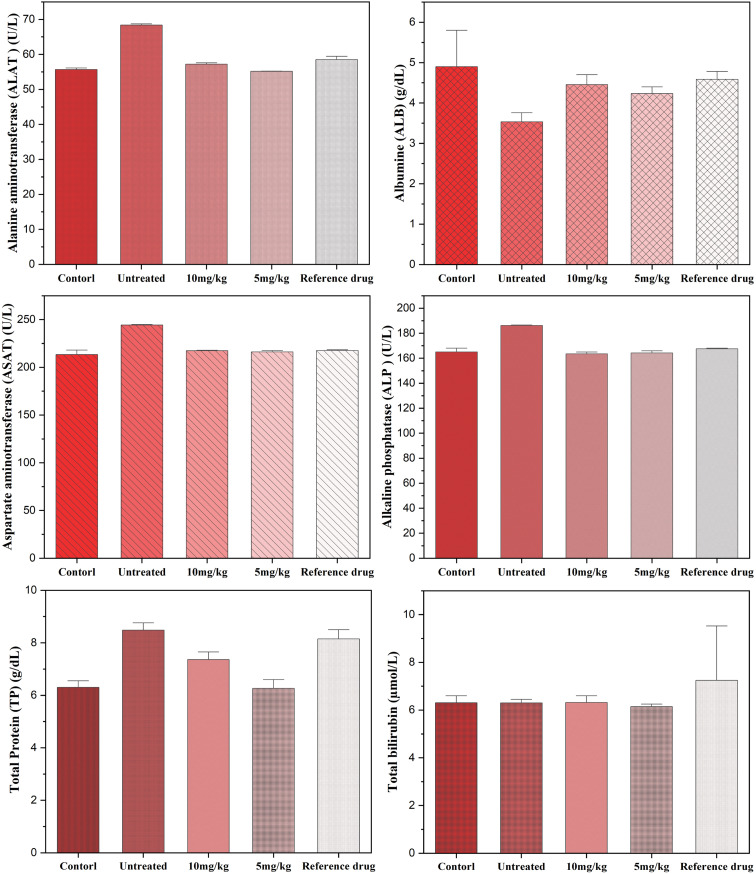
Effect of nanoparticles (10 mg kg^−1^ and 5 mg kg^−1^) on liver function biomarkers.

Regarding hepatic and protein biomarkers, untreated animals exhibited a significant hypoalbuminemia, which was corrected by the administration of 10 mg kg^−1^ and the reference drug, while differences with the 5 mg kg^−1^ dose remained moderate (Fig. 11). These findings are consistent with the observations of Shakil *et al.*,^98^ who demonstrated that biocompatible cobalt ferrites could preserve hepatic protein synthesis. Total protein levels were paradoxically higher in the untreated animals, which may reflect an inflammatory response or a metabolic imbalance, as reported in other experimental models subjected to oxidative stress.^99^ Treatments, particularly the 5 mg kg^−1^ dose, normalized these values, in line with the results of Bentarhlia *et al.*,^100^ who identified this dose as the safest in an integrated toxicological evaluation of Mn–Mg–Co ferrites. Finally, total bilirubin showed no significant variation, suggesting the absence of jaundice or major cytolysis between groups.^101^

Renal function was severely impaired in untreated patients, with elevated creatinine, urea and uric acid levels, three markers commonly associated with renal impairment. These abnormalities were significantly reduced by both doses tested and by the reference drug, with almost complete normalisation of urea and uric acid (*P* < 0.001). These results suggest that treatment with Co_0.5_Mn_0.25_Mg_0.25_Fe_2−*x*_Gd_*x*_O_4_ (*x* = 0.04) has a protective effect on the kidneys, probably by limiting the oxidative stress or inflammation associated with the untreated condition.

**Fig. 12:**
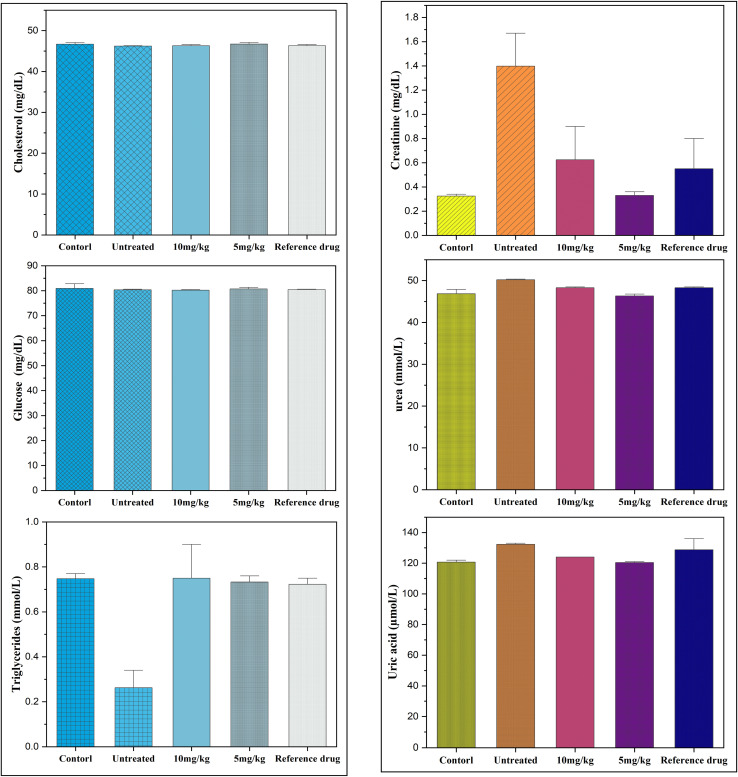
Effect of nanoparticles (10 mg kg^−1^ and 5 mg kg^−1^) on serum metabolic and renal biomarkers.

Metabolically, blood glucose and total cholesterol levels did not differ significantly between groups, suggesting relative stability of carbohydrate and lipid homeostasis in this model. However, triglycerides differed significantly: they were significantly lower in the untreated group than in the treated animals and the reference group, which could reflect exacerbated lipid catabolism in the absence of treatment. Thus, both doses and the reference group appear to restore values closer to normal (Fig. 12).

**Fig. 13:**
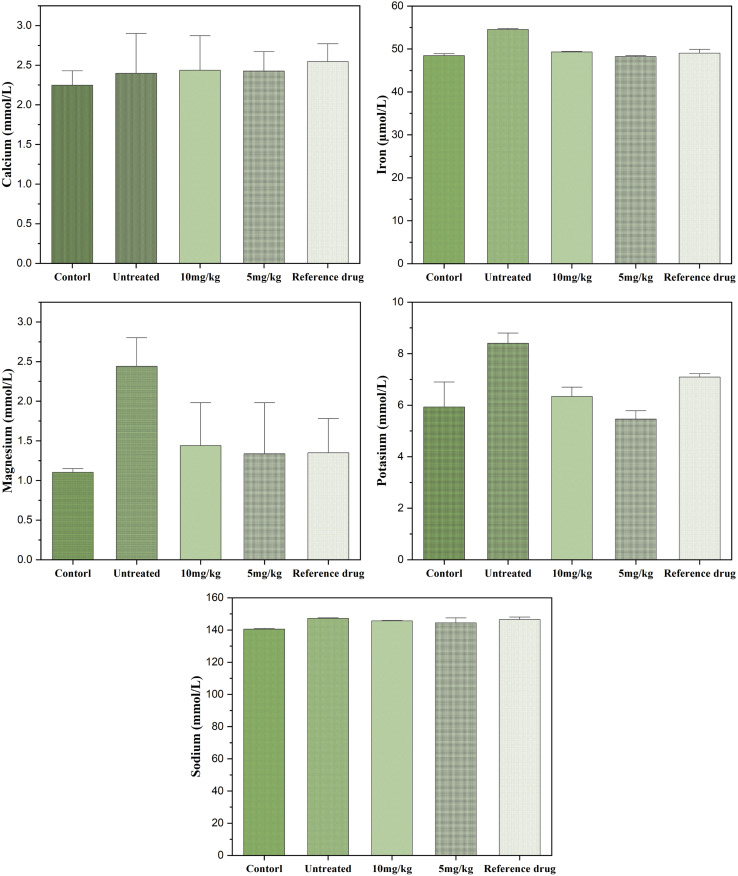
Effect of nanoparticles (10 mg kg^−1^ and 5 mg kg^−1^) on serum electrolytes and minerals.

Electrolyte testing revealed significant disturbances. Sodium levels were significantly higher in untreated patients compared to pre-injection control levels (Fig. 13), indicating a water and electrolyte imbalance that was partially corrected by treatment. Potassium was also elevated in the untreated group, reflecting hyperkaliemia, but the treatments, particularly the 5 mg kg^−1^ dose, brought these values back to physiological levels, sometimes even below those observed with the reference. Calcium and magnesium showed no major differences between groups, with the exception of an increase in magnesium in the untreated group compared to the control, suggesting a temporary disturbance in ionic balance. Finally, serum iron was significantly higher in the untreated group, which could reflect a disturbance in iron metabolism or an increased oxidative state; the treatments effectively reduced this overload, with a more marked gradual normalisation at 5 mg kg^−1^ and 10 mg kg^−1^.

Overall, these results demonstrate that the absence of treatment is associated with significant impairment of numerous biological parameters, including liver function, kidney function, lipid metabolism and electrolyte balance. The two doses tested, comparable to the reference drug, exert an overall protective effect by normalising the majority of the biomarkers analysed. Interestingly, the 5 mg kg^−1^ dose sometimes appears to be more favourable than the 10 mg kg^−1^ dose, suggesting a non-linear dose effect and opening up prospects for dose optimisation. These observations confirm the pharmacological relevance of the molecules studied and their potential in preventing the metabolic and renal disorders observed in untreated conditions.

The results of this study clearly highlight the harmful impact of the absence of treatment on several biological parameters, compared to the groups that received either the reference drug or the tested doses of 5 and 10 mg kg^−1^. Statistical analysis confirms that untreated animals poor nutritional status and inflammation: serum albumin: relationship to inflammation and nutrition consistently show the most unfavourable values, reflecting a state of metabolic, hepatic and renal imbalance. Conversely, the treated groups show significant improvement, with a trend towards normalisation of most biomarkers, suggesting a clear protective effect of the substances tested.

Liver function markers reveal a tendency towards hypoalbuminaemia in untreated patients, corrected by treatment, highlighting the protective effect on hepatic protein synthesis. The absence of significant variations in total bilirubin suggests that no major hepatic cytolysis occurred, but the elevation of total protein in untreated patients may reflect an inflammatory or dehydration state, frequently described in models of oxidative stress. These observations are consistent with other studies reporting that oxidative stress and systemic inflammation can suppress albumin synthesis while increasing acute-phase proteins, leading to hypoalbuminaemia as a marker of poor prognosis, whereas antioxidant or hepatoprotective interventions tend to restore albumin levels and normalize protein metabolism.^102,103^

Renal function appeared to be particularly affected in the untreated group, with significant increases in creatinine, urea and uric acid. These results indicate a reduction in glomerular filtration rate and renal metabolic overload. The significant improvement achieved with the treatments, comparable to that of the reference drug, confirms the nephroprotective effect of the substances tested. The reduction in uric acid is a key indicator, as chronic hyperuricaemia is associated with metabolic and cardiovascular disorders. These protective effects are consistent with observations made with other nanomaterials known for their role in regulating purinergic pathways and reducing renal oxidative stress.

In terms of metabolism, blood sugar and cholesterol levels showed no significant differences, suggesting that the model used does not induce any major carbohydrate-lipid imbalance. However, triglyceride levels were lower in the untreated group, which could reflect increased lipid catabolism in situations of physiological stress. The treatments restored values closer to normal, reflecting a stabilising effect on lipid metabolism. This result is consistent with previous studies demonstrating that certain nanoparticles improve plasma lipid regulation and reduce lipid peroxidation.

Electrolytes and trace elements also highlight the imbalance in the untreated group: hyperkalaemia and hypernatraemia suggest alterations in renal function and water-electrolyte regulation. Treatment corrected these abnormalities, with a particularly marked effect from 5 mg kg^−1^, confirming its regulatory potential. The increase in serum magnesium and iron in the untreated group is also noteworthy: iron overload, in particular, is known to accentuate oxidative stress and tissue damage *via* the production of hydroxyl radicals. The treatments reduced these excesses, suggesting an antioxidant and homeostatic action of the substances tested.

Our results show that at doses of 5 and 10 mg kg^−1^, Co_0.5_Mn_0.25_Mg_0.25_Fe_2−*x*_Gd_*x*_O_4_ (*x* = 0.04) ferrite nanoparticles effectively corrected the biochemical disturbances observed in untreated animals, particularly at the renal level (creatinine, urea, uric acid) and electrolyte balance (K^+^, Na^+^), without significantly altering blood glucose or total cholesterol. These data suggest a favorable biocompatibility profile within the tested dose range, in agreement with studies on other biocompatible ferrites. For example, chitosan-coated CoFe_2_O_4_ nanoparticles demonstrated good tolerance in rats up to 20 mg kg^−1^ intravenously, while simultaneously improving MRI contrast.^98^ Similarly, MnFe_2_O_4_ nanoparticles administered at 3–10 mg kg^−1^ in mice were used as MRI contrast agents without major toxic effects.

Our findings are also consistent with recent work on mixed Mn–Mg–Co ferrites, which indicated that 5 mg kg^−1^ represents a safe dose based on hematological, biochemical, and histological parameters.^100^ This supports the observation that, in our study, the 5 mg kg^−1^ dose was sometimes more effective than 10 mg kg^−1^ in normalizing certain parameters, particularly potassium levels, suggesting the existence of an optimal therapeutic window. Conversely, it is well documented that ferrites administered at very high doses can induce oxidative/antioxidant imbalance and hepato-renal injury, as shown for Ce–Mn ferrites at 1000 mg kg^−1^,^90^ and that toxicity strongly depends on composition, doping, coating, and administration route.^104^ However, certain formulations, such as citrate-coated CoFe_2_O_4_, demonstrated good tolerance even at high doses in rats after prolonged intraperitoneal administration,^105^ underlining the importance of surface chemistry and colloidal stabilization in determining toxicological profiles.

Gd^3+^ doping plays a key role in optimizing magnetic properties: several studies have shown that incorporating gadolinium into the ferrite matrix alters cation distribution and improves hyperthermia performance.^106,107^ Hybrid formulations such as Fe_3_O_4_ Gd have even been administered at 10 mg kg^−1^ in rats for T_2_-weighted MRI, confirming the relevance of this doping strategy for theranostic applications.^108^

Overall, our results confirm that Co_0.5_Mn_0.25_Mg_0.25_Fe_2−*x*_Gd_*x*_O_4_ (*x* = 0.04) ferrite nanoparticles present promising pharmacological and diagnostic potential, capable of correcting the metabolic and renal alterations observed under untreated conditions, with an efficacy comparable to the reference drug and, in some cases, an advantage at the lower dose (5 mg kg^−1^). These findings are in line with previous studies highlighting multicationic ferrites as strong candidates for biomedical applications combining safety and efficacy. Moreover, Mn-doped ZnFe_2_O_4_ ferrites administered at 5 mg kg^−1^ intravenously enabled excellent T_2_ imaging with negligible hematobiological toxicity,^109^ while PEG-coated spinel Zn_*x*_Mn_1−*x*_Fe_2_O_4_ ferrites at 2–8 mg kg^−1^ induced no significant hepatic or renal alterations.^110^ Nickel zinc ferrites (NiZnFe_2_O_4_) tested at 10 mg kg^−1^ intravenously also demonstrated favorable biodistribution and efficient clearance without notable toxic accumulation.^111^

## 4 Conclusion

Co_0.5_Mn_0.25_Mg_0.25_Fe_2−*x*_Gd_*x*_O_4_ (*x* = 0.00; 0.04; 0.06) nanoferrites crystallize as single-phase spinels with controlled cation distributions confirmed by XRD/Rietveld, FTIR and XPS, and exhibit nanometric, composition-uniform particles by TEM/EDS. Gd^3+^ incorporation tunes magnetocrystalline anisotropy and interatomic geometry, yielding a favorable magnetic profile at *x* = 0.04 (*M*_s_ = 45.7 emu g^−1^; Hc = 427 Oe) and efficient magnetic heating (SAR = 34 W g^−1^) compatible with hyperthermia targets. Biologically, the *x* = 0.04 formulation enhances antioxidant status (SOD, CAT) and corrects multiple serum biochemical disruptions (hepatic proteins, renal markers, electrolytes/iron), demonstrating hepatoprotective/nephroprotective potential; antibacterial tests further reveal stronger action against Gram-positive strains. These results position Gd-tuned Mn–Mg–Co nanoferrites especially near *x* = 0.04 as promising, multifunctional candidates for theranostics that combine magnetic hyperthermia with antioxidant hepatoprotection and antibacterial effects. Future work should optimize surface coatings/colloidal stability and investigate dose–response, biodistribution, and long-term safety to accelerate translation.

## Conflicts of interest

The authors declare that they have no conflicts of interest.

## Data Availability

The experimental data supporting the findings of this study (including XRD, FTIR, TEM, and magnetic measurements) are available from the corresponding author upon reasonable request.
